# mHealth in the Wild: Using Novel Data to Examine the Reach, Use, and Impact of PTSD Coach

**DOI:** 10.2196/mental.3935

**Published:** 2015-03-25

**Authors:** Jason E Owen, Beth K Jaworski, Eric Kuhn, Kerry N Makin-Byrd, Kelly M Ramsey, Julia E Hoffman

**Affiliations:** ^1^ National Center for PTSD Dissemination & Training Division Department of Veterans Affairs Palo Alto Health Care System Menlo Park, CA United States; ^2^ Mental Health Services US Department of Veterans Affairs Menlo Park, CA United States

**Keywords:** PTSD, trauma, mHealth, mental heatlh, mobile app, public health, self-management

## Abstract

**Background:**

A majority of Americans (58%) now use smartphones, making it possible for mobile mental health apps to reach large numbers of those who are living with untreated, or under-treated, mental health symptoms. Although early trials suggest positive effects for mobile health (mHealth) interventions, little is known about the potential public health impact of mobile mental health apps.

**Objective:**

The purpose of this study was to characterize reach, use, and impact of “PTSD Coach”, a free, broadly disseminated mental health app for managing posttraumatic stress disorder (PTSD) symptoms.

**Methods:**

Using a mixed-methods approach, aggregate mobile analytics data from 153,834 downloads of PTSD Coach were analyzed in conjunction with 156 user reviews.

**Results:**

Over 60% of users engaged with PTSD Coach on multiple occasions (mean=6.3 sessions). User reviews reflected gratitude for the availability of the app and being able to use the app specifically during moments of need. PTSD Coach users reported relatively high levels of trauma symptoms (mean PTSD Checklist Score=57.2, SD=15.7). For users who chose to use a symptom management tool, distress declined significantly for both first-time users (mean=1.6 points, SD=2.6 on the 10-point distress thermometer) and return-visit users (mean=2.0, SD=2.3). Analysis of app session data identified common points of attrition, with only 80% of first-time users reaching the app’s home screen and 37% accessing one of the app’s primary content areas.

**Conclusions:**

These findings suggest that PTSD Coach has achieved substantial and sustained reach in the population, is being used as intended, and has been favorably received. PTSD Coach is a unique platform for the delivery of mobile mental health education and treatment, and continuing evaluation and improvement of the app could further strengthen its public health impact.

## Introduction

Smart mobile devices are increasingly ubiquitous, discreet (ie, can be used in public spaces without attracting attention), convenient (ie, carried at all times, immediate access to services), capable of running powerful software applications or “apps” that collect data and respond to user actions, and are highly functional allowing users to easily access services, such as banking, getting directions, and connecting with friends. Approximately 55% of adults in the United States own mobile phones, and 42% own tablets [1]. The use of apps among mobile device owners has increased from 22% in 2009 to 50% in 2013; increasing at a rate that parallels the early rapid growth of Internet use [1]. In 2014, accessing the Internet via mobile devices surpassed Internet access via personal computers (47% compared to 45%).

Mobile health (mHealth) refers to an interdisciplinary branch of eHealth, focused on utilizing mobile technologies for health improvement initiatives [2,3]. Because of the widespread adoption of mobile devices, mHealth technologies have the potential to reach large numbers of individuals in the general population, including those who are living with untreated mental health concerns and as a supplement to those already in care [4]. Because many of those with posttraumatic stress disorder (PTSD) do not receive evidence-based treatment [5], PTSD is an important candidate for testing mobile mental health technologies. In the United States, the lifetime prevalence of PTSD is estimated to be between 7 and 12% of the population, and between 5 and 7 million adults are living with PTSD in any given year [6]. Despite the high prevalence of PTSD, seeking mental health treatment, particularly among veteran populations, is stigmatized [7,8]. Mobile mental health apps have the potential to provide mental health support that overcomes barriers posed by stigma, and technological interventions for PTSD may provide helpful resources to individuals who would not otherwise seek traditional face-to-face care.

Emerging research on the usability, feasibility, and efficacy of mHealth interventions demonstrates promise [3,9]. However, extant mHealth studies have been constrained by small sample sizes, use of private research versions of apps that are generally unavailable to the public, use of reimbursement to incentivize participation, and limited evaluation of user experiences with the technology. In contrast, few mHealth apps that have been successfully disseminated to the general public have also undergone scientific evaluation. To our knowledge, there are no studies that have evaluated mobile mental health technologies deployed in real-world settings. Thus, little is known about natural patterns of engagement, optimal design features, user experiences, and the most effective applications of mobile phone technologies. Maximizing the efficacy of mHealth interventions for PTSD will require a better, more ecologically valid understanding of how apps for mental health are used by the general population.

**Figure 1:**
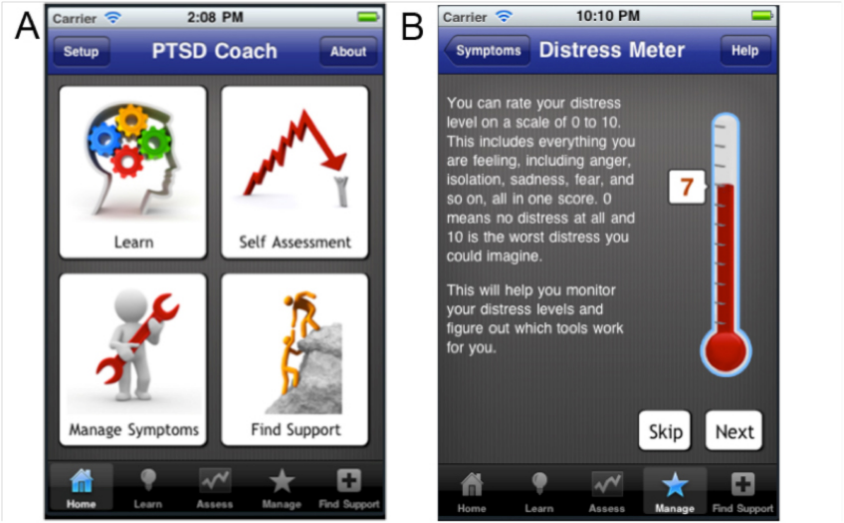
Screenshot of the PTSD Coach mobile phone application: (A) home screen and primary content areas: Education (Learn), Self-Assessment, Manage Symptoms (Tools), and Support; (B) Distress Thermometer (ie, subjective units of distress, measured before and after use of a symptom management tools).

Preliminary research on the utility of mHealth for PTSD suggests that a mobile intervention would be well-received by veterans and useful for managing symptoms such as anxiety and sleeplessness or during periods of intense stress [10]. To meet this need, the United States Department of Veterans Affairs (VA), in collaboration with the Department of Defense, developed PTSD Coach, a mobile mental health app for PSTD, for both iOS and Android operating systems [11,12]. The app is designed for veterans, service members, and civilian trauma survivors who may be experiencing symptoms of PTSD, as well as for their family members and others desiring to learn about posttraumatic stress. The design of PTSD Coach was informed both by subject matter experts in evidence-based treatment of PTSD and Veterans and others living with PTSD [10]. The app provides authoritative information about PTSD and professional care, a self-assessment for PTSD symptoms, opportunities to find support, and cognitive-behavior therapy (CBT) based interactive tools to help users manage PTSD symptoms (see Figure 1).

Since its release in early 2011, PTSD Coach has been widely available to the general public [11,12], thereby providing a unique opportunity to evaluate the potential public health impact of a mobile mental health app for PTSD. Two sources of data shed some light on how PTSD Coach has been received in the general population. First, aggregate and fully de-identified data are available to describe how users in the general population interact with PTSD Coach. Aggregate app usage data can be very useful for understanding patterns of attrition and how users interact with key content or features. Such information is crucial for optimizing user experiences and tailoring the delivery of evidence-based intervention components of the app. Second, many users provide thoughtful written reviews of the app, and a detailed qualitative analysis has the potential to greatly inform developers about how users engage with mobile mental health apps and opportunities for further enhancing their efficacy.

By combining quantitative and qualitative data sources, mixed methods has the potential to increase both the breadth and depth of our understanding of mHealth interventions being delivered in uncontrolled, real-world settings [13]. Qualitative data allow for a richer understanding of the processes by which an intervention may exert its effects [14], and are particularly valuable for understanding how interventions are perceived by users, whether they are used as intended, and what underlying, unmeasured factors might influence adoption and use. Mixed methods also provide a means for app users to have a voice in informing the development of the population-based mobile mental health apps.

The current study characterizes reach, use, and impact of PTSD Coach in the general population using a mixed-methods approach. Two distinct types of data were analyzed: aggregate population-level app engagement data and qualitative data from user reviews in the two major app marketplaces: Apple’s App Store and Google’s Play Store. The study had three aims: (1) to examine the reach of PTSD Coach over time; (2) to characterize how users engaged with the app; and (3) to describe the reception and impact of the PTSD Coach mobile app among users in the general population. We also sought to investigate potential differences between iOS (Apple) and Android versions of PTSD Coach.

## Methods

### Data Sources

Data were derived from two sources. First, we included mobile analytics data from all unique downloads of PTSD Coach in the three-year period between March 2011 and February 2014 (N=153,834). Detailed session event logs from March 2014 through June 2014 were also available (N=3462 first-time sessions; N=12,449 return visit sessions). Second, we included written reviews from all PTSD Coach users (N=156) who provided reviews of the app on either the App Store (Apple) or the Play Store (Google).

### Procedures

#### Mobile Analytics

User engagement with the app was measured using Flurry Analytics. The Flurry software development kit (SDK), with a restricted feature set to ensure de-identification, was integrated into the iOS and Android versions of PTSD Coach prior to their public release in order to be able to make administrative decisions about how to deploy the app for the most public health impact while simultaneously ensuring user privacy and confidentiality. To accomplish this balance, VA opted to use Flurry Analytics to obtain de-identified, aggregate data about how users interact with PTSD Coach. No identifying information of any kind was available for any user of the app, and information across sessions could not be tracked. Flurry was used to securely capture aggregate usage data and retention information across time, as well as to capture key app-related events (eg, agreeing to end user license agreement or EULA, viewing home page, visiting app content). All usage measures were stored in aggregated, anonymized data files and were available separately for the iOS and Android versions of the app.

#### User Reviews and Ratings

All user reviews provided for PTSD Coach between April 8, 2011 and February 10, 2014 were obtained from the App Store (Apple) and Play Store (Google), where they are publically available. Two autotranslated and unintelligible user reviews from the Play Store were removed from the sample. The final sample consisted of 156 user reviews (App Store, n=53; Play Store, n=103). In order to write reviews in the App Store, users are first required to download the app. For the Play Store, users can review an app without downloading it. Reviews are not required, nor does PTSD Coach prompt users to write reviews.

In addition to user reviews, star ratings were also captured. All app users were provided with the option to rate PTSD Coach using a 5-star ratings system that is common across platforms (ranging from 1 to 5, with 1 being the most negative rating, and 5 being most positive). Users may leave a “star” rating for the app without providing a written review. All star ratings were analyzed, regardless of whether the user left an app review or not (*n*_iOS_=80, *n*_Android_=329). User reviews and ratings were imported into SPSS 21 for analysis.

### Measures

#### Mobile Analytics

For each platform (iOS or Android), we captured basic user engagement measures (ie, number of downloads, active users, session length, number of sessions, etc across time) in addition to two primary measures of retention across time. Retention was first measured using *rolling retention,* which is the ratio of the number of users whose last day of app use was some time after a given time point to the total number of users who downloaded the app. Because rolling retention does not account for the frequency of use and may overestimate actual usage, we also measured retention using *return rate,* which captures the proportion of users who returned to use the app during specific intervals of time (eg, one day after download, the first week after download, one month later, etc). Finally the Flurry mobile analytics package also allowed us to capture *fully de-identified* click stream data*.* Click streams, which documented users’ navigation through the content pages of the app across time, were parsed using the Perl programming language to identify first-time users (those who accepted the EULA) and returning users. Each session began with the launch of the app and was classified as either a first-time use or a return visit on the basis of whether the EULA was displayed at launch or not. For each session type (first time use versus return visit), specific usage events were tracked, including completing set-up of the app, navigation from the home screen to one of the four primary content areas (learn, manage symptoms, find support, or self-assessment) and navigation from one content area to another. Click streams were only available for the iOS version of the app. Given the high volume of click stream data, we analyzed a representative subset from the most recent three month of app use, which comprised over 650,000 rows of data.

The PTSD Coach app was also able to capture self-report data on PTSD symptoms and momentary distress. PTSD symptoms were measured using the PTSD Checklist-Civilian version, or PCL-C [15]. The PCL-C is a widely-used 17-item self-report measure of PTSD symptoms that has strong reliability and validity [16,17]. Upon visiting the self-monitoring section of the app, participants were prompted to complete the PCL by rating each of the 17 PTSD symptoms on a 5-point Likert scale ranging from 1 (not at all) to 5 (extremely), with total scores ranging from 17 to 85. Momentary distress was measured using a one-item Subjective Units of Distress Scale (SUDS, or distress thermometer [18]), anchored at 0 (no distress) and 10 (worst distress you can imagine). This measure has been widely used in PTSD research and validated against more extensive measures of mood disturbance in a variety of populations [19].

#### Qualitative Codes From User Reviews

In order to characterize users’ personal experiences with the app, a phenomenological approach to qualitative analysis was utilized. Three domains of interest, based on our primary research questions, were identified for qualitative coding: app reach (eg, characteristics of individual app users), usage (eg, how and when users interacted with the app), and reception (eg, perceived outcomes associated with using the app and the overall valence of users’ impressions of the app). Separate coders (J Owen & B Jaworski) categorized each user review for the presence or absence of each broad coding domain. Consistent with an inductive approach to qualitative analysis, coders identified specific themes within each domain, and careful coding rules were used to create a codebook (available from the authors) to characterize each of these themes. User reviews were then coded independently by the two coders, and inter-coder reliability demonstrated substantial to near-perfect agreement (kappas=.65-.93, mean=0.77; [20]). All coding disagreements were resolved by consensus coding. Overall, user reviews were brief with a mean value of 32.8 words (SD 32), averaging just over two assigned codes per review with a mean of 2.2 (SD 2.0). iOS user reviews were longer with a mean value of 53.2 words for iOS versus 44.3 for Android (*t*_60.6_ =4.87, *P*<.001), and were assigned more codes than were Android reviews, with a mean of 3.6 for iOS versus 1.5 for Android (*t*_66.8_=6.37, *P*<.001).

### Analyses

To examine the reach of PTSD Coach, we calculated descriptive statistics and proportions for all basic user engagement metrics from the Flurry mobile analytics package. For some aggregate metrics, standard deviations could not be estimated when individual data points were not available for analysis (eg, session length, number of total sessions using the app). Qualitatively identified characteristics of users who left app reviews were also summarized. To characterize usage of the app, we tested differences between iOS and Android users with respect to completing key app-related tasks (ie, completing EULA, engaging in app set-up, viewing one of the four primary content areas on the app, and viewing multiple content areas). Differences between platforms were tested using chi-square analyses. Differences in app ratings between platforms were tested using an independent samples *t* test. To evaluate reception, proportions of iOS and Android users expressing each qualitatively identified code or valence were compared using chi-square analyses. To evaluate impact, change scores for momentary distress were calculated and tested, using a one-sample *t* test against no change. All analyses were conducted using SPSS 21.

## Results

### Reach of PTSD Coach

**Figure 2:**
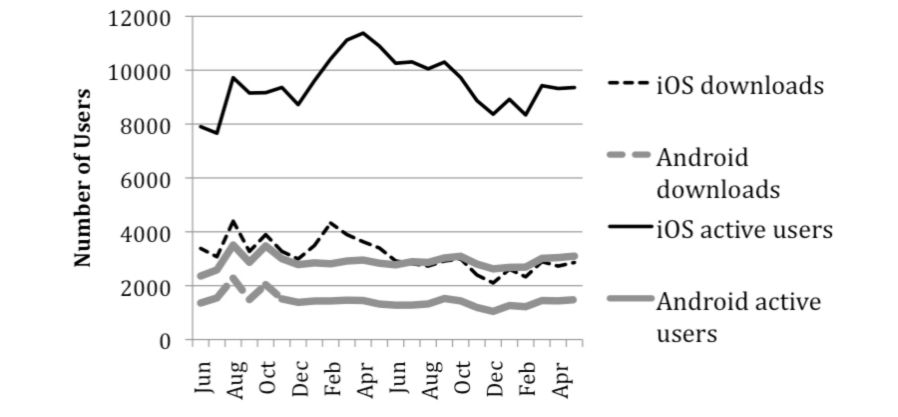
Downloads and active users of PTSD Coach between June 2012 and May 2014.

Since its launch in the spring of 2011, PTSD Coach has been downloaded 153,834 times, and 64% of these downloads were on the iOS platform. The number of new downloads has been relatively stable across time, with over 55,000 in 2012 and nearly 54,000 in 2013. There was an average of over 10,600 unique active users in any given month (ie, used at least once during the month) since the release of the app. Figure 2 displays number of downloads and active users for the previous two years (ie, June 2012 – May 2014). As of September 2014, PTSD Coach has been downloaded in 86 countries, with non-US downloads constituting 12% of the total and 10-15% of each month’s new users.

PTSD Coach was intended for a wide range of individuals impacted by trauma, not just those who have served in the military. Qualitative results suggest that PTSD Coach is reaching veterans and civilians, as well as family members and mental health providers. Personal characteristics, such as military service, PTSD diagnosis, and psychotherapy attendance, were mentioned in 39 (25%) of the reviews. Among those who disclosed military status (n=22), 73% self-identified as current service members or veterans, compared with 27% who self-identified as being civilians. Of those who mentioned trauma characteristics (n=19), all users reported trauma exposure or a specific PTSD diagnosis (eg, “I suffer from PTSD”). A minority of user reviews (n=9) mentioned being connected to someone who had experienced trauma, such as family members (eg, “My spouse has PTSD”) and mental health service providers (eg, “I’m a counselor who hopes to use this app with clients”). PTSD Coach also captured self-reported PTSD symptoms (using the PCL) for 10.3% of first-time users’ sessions and 20.9% of returning users’ sessions. Mean PCL scores, in first-time sessions was 57.2 (SD 15.7) and in return sessions was 55.1 (SD 16.6), which were well-above the National Center for PTSD’s recommended probable PTSD diagnosis cut point for use in VA or civilian specialty mental health clinics [21].

### Use of PTSD Coach

#### Retention of Users

Mobile analytics data were used to identify patterns of app use across time. First, we examined rolling retention rates which describe the proportion of users who continued active application use across time. Among those who downloaded PTSD Coach (n=153,834), 61.1% returned to use the app after the first day it was installed. Over half of users (52.1%) continued to use the app, or used it at least one time beyond the first week of download. Usage declined over time, with 41.6% continuing to use the app 1 month after installation, 28.6% using the app after 3 months, 19.4% using the app after 6 months, and 10.6% using the app a full year post-installation. Patterns of user retention varied based on operating system, with iOS users maintaining both a higher number and proportion of users across time.

**Figure 3:**
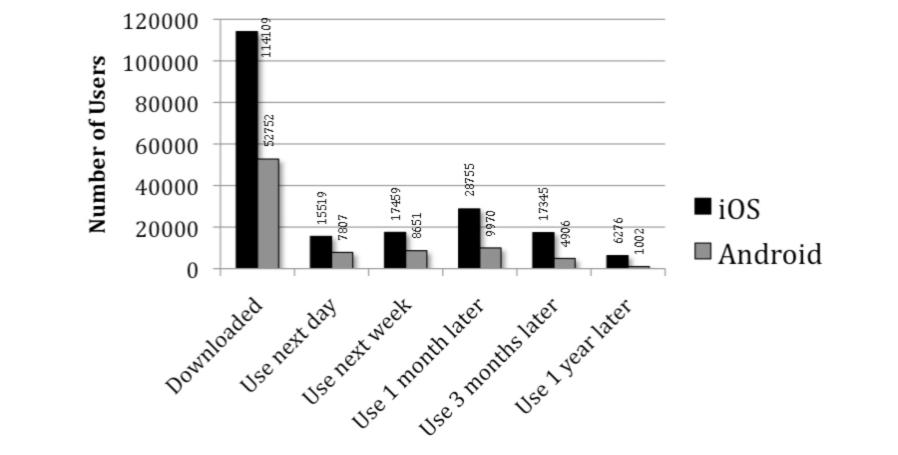
PTSD Coach use and maintenance up to one year after initial download.

Because rolling retention metrics include users who may use the app infrequently or sporadically across time, we also identified actual app usage per user during specific time periods post-download (see Figure 3). Use of PTSD Coach during the day and week after initial download were quite similar for iOS and Android, averaging 14% of users who opened the app one day after download and 15.6% of users who opened the app sometime within the first week after the initial download. By one month after initial download, iOS and Android return rates began to differ, with 25.2% of iOS and 18.9% of Android downloaders returning to use the app sometime within that first month. During the third month after download, 15.2% of iOS and 9.3% of Android users returned to use the app. By one year post-download, 5.5% of iOS and 1.9% of Android users returned to use the app.

Analysis of user reviews identified few mentions of use patterns or habits (n=15) but suggested discrete subsets of users: those who used PTSD Coach only once or a few times and those who used PTSD much more extensively. Overall, 6.4% (n=10) of user reviews reported that they used the app during moments of crisis or described the app as being available exactly when it was needed. Another 4.5% (n=7) of reviews described adoption of the app into the user’s daily routine (eg, “I have used this app at least once a week”) and long-term use (eg, “I have used this for at least a year now”). Self-reported momentary distress levels, reported by 12.7% of those in a first-time session and 20.2% of those in a return-visit session, were consistent with qualitative findings suggesting use during moments of need. Distress levels in both first-time (mean 6.7 [SD 2.1]) and return-visit sessions (mean 7.0 [SD 2.1]) were quite high, and return-visit users exhibited higher momentary distress levels than first-time users, *t*_2956_=2.76 , *P*=.0057.

#### Characterizing App Sessions

Most app usage occurred between 8am and 10pm (of the user’s time zone), with an average of 30.2 users per hour over a 24-hour period, peaking at 1pm (with a mean value of 43 users/hour) and declining steadily until 10pm (with a mean value of 32 users/hour). Usage between the hours of 11pm and 6am averaged nearly 19 users per hour. The median number of sessions per user was 1.7 per day, 1.9 per week, and 2.5 per month. On average, users opened PTSD Coach (ie, began a new session) 6.3 times before discontinuing use. Median time spent using PTSD Coach during each session was just over 47 seconds, and total time spent using PTSD Coach averaged approximately 5 minutes (see Table 1 for details, by platform). The app was used throughout the day, including times outside typical clinic business hours. User reviews complement these findings and highlight the strength of mHealth solutions for providing in-the-moment access. For example, one review described how the app helped during difficult times, stating that

It helps get me through those rough patches, and when you’re really messed up it will advise you to call someone in your support line.

Another user highlighted the availability of the app’s resources, commenting that

[it was] great to be able to carry all this around in my pocket. When I can't sleep or I feel like I'm going to lose it, I can't always wait to see a counselor.

**Table 1:** Aggregate Flurry measures of app usage and App Store satisfaction ratings by platform.

Flurry measures	iOS	Android	Total
Mean # of sessions per user (mean)	6.8	5.2	6.3
Median time per session (seconds)	47.8^a^	47.6^b^	47.7
Mean total time of use (seconds)	325	250	301
Mean App Store rating^a^	4.5	3.9	4.3

^a^App store ratings are based on a 5-star system where 5 stars is the highest possible rating that can be assigned; app ratings represent only a subset of total downloads with n=80 for iOS and n=329 for Android; Total app rating is weighted by number of downloads.

^a^Average for all health & fitness mobile apps tracked by Flurry for iOS=39.9 seconds

^b^Average for all health & fitness mobile apps tracked by Flurry for Android=31.7 seconds

Click stream data were analyzed for the most recent three months of user sessions with PTSD Coach in order to provide more detailed information on differing usage patterns between first time and returning users (see Table 2). When first using PTSD Coach, the app requires users to agree to the EULA, and then users may complete an optional brief set-up to customize the app with personal data and media from their phone (eg, music, photos, contacts). Users who opt to skip this set-up go directly to the app’s home screen which presents the four main content areas of the app (see Figure 1). After the app was opened, the majority of first-time users arrived at the home screen (79.8%) and 37% visited a content area. In contrast, among returning users, 63.3% navigated to at least one content area. Among first-time users who made it to a content area (n=1275), 32.5% accessed one content area, 43.6% accessed two or three of the content areas, and 24.7% accessed all four content areas of the app. The majority of returning users navigated directly to the assessment (61.7%) or symptom management (53.4%) sections of the app, and only 44.5% of those who viewed content visited more than one content area.

**Table 2:** Detailed session analysis for PTSD Coach (iOS) by first time users and returning users: March 5, 2014 through June 14, 2014.

Session analysis		First time users (n=3462 sessions)	Returning users (n=12,449 sessions)	Between-group differences (*P* value)
Number of events within session, mean (SD)		31.2 (42.0)	29.4 (36.8)	.01
Consented to license agreement, n (%)		3383 (97.7%)	-	
Entered app set-up, n (%)		1879 (54.3%)	1187 (9.5%)	<.001
Made it to home screen, n (%)		2762 (79.8%)	12,418 (99.8%)	<.001
Made it to any content area, n (%)		1285 (37.1%)	7882 (63.3%)	<.001
**First content area visited** ^a^ **, n (%)**				<.001
	Learn about PTSD	437 (34.0%)	1185 (15.0%)	
	Self-assessment	409 (31.8%)	3597 (45.6%)	
	Manage symptoms	266 (20.7%)	2399 (30.4%)	
	Get support	173(13.5%)	701 (8.9%)	
**Each content area visited within session** ^a^ **, n (%)**				
	Learn about PTSD	773 (60.2%)	2308 (29.3%)	<.001
	Self-assessment	870 (67.7%)	4860 (61.7%)	<.001
	Manage symptoms	821 (63.9%)	4210 (53.4%)	<.001
	Get support	590 (45.9%)	2534 (32.1%)	<.001
**Number of content areas visited** ^b^ **, n (%)**				<.001
	0	1477 (53.5%)	4536 (36.5%)	
	1	414 (15.0%)	4368 (35.2%)	
	2	288 (10.4%)	1779 (14.3%)	
	3	268 (9.7%)	954 (7.6%)	
	4	315 (11.4%)	781 (6.3%)	

^a^denominator=all those who made it to at least one content area on the app; ^b^denominator=all those who made it to the app’s home screen.

Qualitative data provided additional information about users’ likes and dislikes associated with each of the app’s main components. Across all reviews, 27.6% (n=43) described one or more specific features of the app that they particularly liked. Largely consistent with the mobile analytics findings, users most commonly reported liking the manage symptoms tools (13%), followed by self-assessment (9%), learn (6%), and find support (<1%). Fewer than 10% (n=15) described specific dislikes, and those dislikes centered on personal preferences about the manage symptom tools and self-assessment. For example, one user did not like the pace of the breathing tool, noting “I'd like to breathe slowly, not hyperventilate” and another user indicated that the self-assessment did not capture data over a sufficient length of time stating:

been using [this app] for over a year now to take self-assessments, but can't view history of those over this longer span of time.

However, 33.3% (n=52) reported at least one technical problem. Android users noted a high prevalence of technical problems (46.6%) relative to iOS users (7.5%; χ^2^_1_(N=156)=24.0, *P*<.001). Over 15% (n=24) of reviews mentioned a specific suggestion for improvement, such as providing a feature for social networking with other users, creating specific web and tablet versions of the app, providing an ability to journal on the app, and providing features that would be used in conjunction with a professional therapist.

### Reception and Impact of PTSD Coach

Reviews were predominately classified as having a positive valence (58.3%, n=91), having a negative valence (25.6%, n=40), or being coded as ambivalent between positive and negative (16.0%, n=25). Valence of reviews differed significantly between iOS and Android platforms, χ^2^_2_(N=156)=31.2, *P*<.001, with 89% of iOS reviews classified as entirely positive compared with 42.7% of Android reviews. Similar to the valence of the written reviews, star ratings among those who provided a review indicated favorable views of the app, with an average of 3.6 stars across both platforms, with significant differences between iOS (mean*=*4.6, SD=0.9) and Android (mean=3.1, SD=1.7; *t*_154_=7.6, *P*<.001).

From the qualitative analysis, seven categories of perceived meaningful outcomes were identified and coded (see Table 3). The majority of reviews indicated that PTSD Coach has a

**Table 3:** Perceptions of meaningful outcomes associated with use of the PTSD Coach mobile app from user reviews.

Reported outcomes associated with app use	Example review comments
General helpfulness or usefulness of the app	“*This is very effective & helpful.”; “If you had to deal with trauma this is exactly what you want. To be able to help yourself deal with things better”*
Gratitude for the app	“*Thank you for all your efforts in providing such a supportive app”; “I am thankful to have it”*
Gratitude or appreciation specifically toward the VA	“*Thanks to the workers at the VA for trying so hard to help us”;* “*Really great that the VA made available a mobile app to assist with treatment of PTSD”*
Meaningful personal difference	“*This app took me from severe to moderate PTSD!”;* “*Instead, I was able to use a tool on the app for relaxation. The flashback subsided and I was able to get right back to sleep.”*
Life-changing or life-saving impact	“*This app just might save a few lives. I know it saved mine”;* “*This has changed how I deal in private and a god send”*
Increasing or reinforcing face-to-face PTSD treatment or serving as a primary tool for self-management	“*I found out it’s useful for them in tailoring you treatment”; “Reminding me of the skills I know, awesome!”*
Adverse or negative experiences	“*Frustrating”;* “*Stresses me out!”*

positive impact on its users. Nearly one-third of reviews (n=48) explicitly described the app as being helpful (50.9% of iOS users, 20.4% of Android users). Nearly 15% (n=23) of reviews expressed direct gratitude for the app (30.2% of iOS user reviews and 6.8% of Android user reviews), and 12.2% (n=19) reported that the app made a meaningful personal difference or had a life-changing impact (26.4% for iOS users, 4.9% for Android users). Only one user indicated feelings of increased stress or anxiety, which was attributed to technical difficulties using the Android version of the app.

For both first-time and return-visit sessions, 21% of users who provided a momentary distress rating also went on to use a suggested symptom management tool and then re-rated their distress. Momentary distress scores decreased an average of 1.6 points (SD=2.6; 95% CI=1.07–2.14) points for first-time users, which was statistically significant, *t*_92_=5.0, *P<*.001 (see Table 4). Among return users, distress decreased an average of 2.0 points (SD=2.3; 95% CI=1.81–2.19), which also was statistically significant, *t*_547_= 20.4, *P*<.001. For the Android platform, n=6125 sessions were available for analysis, and of these SUDS change scores were available for 20.6% (n=1262) of sessions. Average SUDS reduction was 1.14 points (SD=2.35; 95% CI=1.011–1.27), a statistically significant decrease, *t*_1261_=17.2, *P*<.001. SUDS change scores were significantly higher for first-time iOS users than for Android users, *t*_4722_=5.5, *P<*.001.

**Table 4:** Trauma symptoms, momentary distress, and distress reduction associated with use of symptom management tools in PTSD Coach.

		First-time sessions (N=3462 unique users)	Return-visit sessions (N=12,449 sessions aggregated across users)	Between-group differences (*P*value)
**Trauma symptoms (PCL)**				
	Mean (SD)	57.2 (15.7)	55.1 (16.6)	.024
	n (%)	359 (10.3%)	2599 (20.9%)	
**Momentary distress (SUDS)**				
	Mean (SD)	6.7 (2.1)	7.0 (2.1)	.005
	n (%)	440 (12.7%)	2518 (20.2%)	
**Reduction in distress (SUDS) after use of symptom management tool**				
	Mean reduction (SD)	1.6 (2.6)	2.0 (2.3)	.13
	n (%)	93 (21.1%)^a^	548 (21.8%)^a^	

^a^denominator=all those who provided at least one momentary distress rating; Time frame: March 5, 2014 through June 14, 2014. n=proportion of users who entered self-report data; SUDS=Subjective Units of Distress.

## Discussion

The public health impact of an intervention can generally be characterized as a function of its reach and efficacy [22]. With respect to reach, PTSD Coach has been broadly disseminated, and based on user reviews it appears that a substantial proportion of the users represent its target audience of veterans and civilians in the general population living with PTSD symptoms. Remarkably, in spite of the lack of marketing to promote app use, the reach of the PTSD Coach app has been consistent across time-with more than 50,000 downloads per year in each of the 3 years since its release with a growing international reach. Given its success, the Veterans Affairs departments of Australia and Canada have published their own versions of PTSD Coach, and a Danish version is in production. These numbers demonstrate the potential of PTSD Coach and other mobile apps for reaching a number of specific target populations across the globe, including family members, those with diverse types of trauma-related stressors, those working with and without a therapist, and veterans and civilians. Mobile mental health apps have substantial potential to expand the reach of mental health support services, and PTSD Coach is successfully reaching many with high levels of PTSD symptoms.

Estimates of the use of PTSD Coach provide useful indicators of the app’s potential efficacy by helping us to characterize whether users are engaging with intervention content in a manner that is consistent with its intended use. PTSD Coach was explicitly designed to be used as-needed, during moments of distress, and distress data clearly demonstrate that many users of PTSD Coach were moderately to highly distressed at the time they chose to use a symptom management tool. App use patterns, as identified through both qualitative and quantitative data, were strongly consistent with this design and demonstrate relatively strong use over time, with users averaging just over 6 sessions with the app. First-time and return users also engaged with the app very differently, with return users more readily accessing evidence-based content, such as the self-management tools and self-monitoring intervention.

Additionally, these are among the first data to provide usage and attrition data from a publically available and widely disseminated mHealth application and offer a useful benchmark for evaluating other mobile mental health apps. As with many eHealth technologies [23], developers of mHealth applications must contend with considerable attrition across time. There were subsets of users, identified by both mobile analytics and user reviews, who endorsed sustained, long-term use of the app. In Web-based interventions, greater doses of intervention are associated with stronger effects [24,25], but very little is known about engagement with mHealth tools. Understanding the relationship between engagement and effectiveness of mHealth applications, and how these factors interact with individual characteristics of the user, is a critical next step. It may be that a few well-timed doses of PTSD Coach, accessed when needed most, may be sufficient to have an impact on PTSD-related symptomatology.

Detailed mixed-method analyses of session visits and user experiences also allowed us to identify a number of ways the app could be improved. Importantly, over half of first-time users who made it to the app’s home screen abandoned their sessions before visiting any of the primary content on the app. Among developers, this is a fairly well-known use pattern (downloading an app and quickly scanning it to determine its personal relevance), and many of these users will establish more familiarity with the app by returning for subsequent sessions and continuing to use the app on an ongoing or as-needed basis. For those users who may only open the app a single time, there is only a brief opportunity to capture their interest. Identifying interventions that are carefully tailored to specific subsets of users, such as first-time or infrequent users, could increase immediate adoption and promote sustained, appropriate use of the app. A/B or multivariant testing, whereby two or more versions of the same app are made available to test one specific change to the app (eg, modifying the welcome sequence to encourage rapid access to app content), could be particularly valuable, for example, by helping first-time users successfully navigate to at least one evidence-based content area of the app [3]. Although we have now received detailed qualitative feedback from a face-to-face group of inpatient veterans [10] and online user reviews of the app, additional detailed qualitative interviews, with specific target audiences such as PTSD Coach users with trauma who are not already connected with mental health care or survivors of military or other sexual traumas, could also be used to further strengthen the efficacy and impact of the app for these important subgroups. Additionally, the self-assessment and manage symptom domains were the most heavily used content areas of the app, and efforts to personalize and tailor content to specific user characteristics (eg, trauma severity, distress level, or history of use of the app), could promote repeated or sustained use of the app among those in need [26].

With respect to impact of PTSD Coach, qualitative evaluations of the app were predominately positive, and the perceived helpfulness of the app was among the most commonly identified themes in the study. A substantial number of users reported life-altering benefits, and we observed no reports of any adverse events. Satisfaction ratings were also very strong, particularly among iPhone users. These results highlight that PTSD Coach is well-tolerated and delivers meaningful personal benefits to a number of users. Both first-time and return-visit users who used a symptom management tool and reported momentary distress levels before and after exhibited a significant, modest reduction in distress–approximately 2 full points on the 0-10 scale. The symptom management tools were equally beneficial for first-time and return-visit users. Whether the app can be shown to provide benefit greater compared to passage of time or an active placebo control condition remains an open question.

Platform-related differences in users’ experiences with PTSD Coach (ie, iOS vs Android) may be of particular interest to other mHealth researchers and developers, given the challenges and costs of developing apps for two distinct platforms. As described in many user reviews, the Android version of the app provided a qualitatively different user experience than did the iOS version, and Android users did not experience as much distress reduction from symptom management tools as did iOS users. Because Android is used by 28% of all mobile phone users (compared with 25% for iPhones; [1]) it has the potential to reach a substantial number of those living with PTSD symptoms. However, the costs of developing for Android will be higher because of the need to accommodate the diversity of the Android ecosystem, including multiple devices with widely divergent characteristics and various current and former versions of the operating system itself [27]. For mHealth researchers and developers, it is important to recognize that the choice of platform is critical given the substantial investment of time, energy, and capital required for startup, development, and maintenance of mobile mental health interventions. Some degree of platform specialization may be necessary for the field until better cross-platform mobile development tools become available, particularly those that can satisfy broad dissemination requirements (eg, full compliance with Americans with Disabilities Act accessibility standards).

There are a number of study limitations that must be considered. First, use of the Flurry mobile analytics package did not allow us to identify users at the individual level, and results could only be evaluated in aggregate. This aggregation could actually lead to less accurate estimates of engagement, because mobile analytics overestimate the number of unique downloads by including those who download the app multiple times or on multiple devices. Aggregation also resulted in a potential underestimate of app usage by return users in particular, because interrupted interactions with PTSD Coach (eg, switching back and forth between apps) could not be linked together. Consequently, many such return-visit sessions were not available for analysis. Future studies of PTSD Coach in controlled trials will be required to evaluate how specific users engage with the application over time and how individual differences in PTSD-related factors might impact use. Second, qualitative results were limited to those who chose to leave a user review on one of the app stores, and little is known about whether such reviews are representative of all those who have downloaded and tried the app. Previous research [28] has suggested that earlier reviews may be more positive than later reviews, or that those with more neutral attitudes may be less likely to leave reviews. However we did not observe any time effects associated with valence of the reviews. Additionally, star ratings for PTSD Coach did not differ between those who left reviews and those who did not. Given the potential for bias in the user reviews, additional sources of phenomenological data (eg, detailed qualitative interviews) may be helpful for triangulating the present findings.

PTSD Coach has clearly been helpful to a number of users, and many of those who use the symptom management tools report significant reductions in distress. A subset of users report high levels of use of PTSD Coach and/or life-changing impacts, whereas for others the app appears to meet expectations but is used only a handful of times before being discontinued. Whether the app is able to exert a meaningful effect after only a few sessions is a question that we are currently investigating in controlled studies. This study is among the first to provide such a highly detailed examination of reach, use, and reception associated with a free and publically available mHealth app, and results may serve as a useful benchmark for the evaluation of subsequent apps for mental health. These benchmarks could provide useful targets for effectively improving mobile mental health apps, for example, by reducing next-day or next-week attrition, increasing the proportion of those who access key content during the initial session with the app, and identifying ideal minimum exposures to specific content areas that would be considered necessary to exert a beneficial effect. Given the broad dissemination of PTSD Coach, even minor efforts to further refine the usability and utility of the app may ultimately increase the number of those with traumatic stress symptoms who receive a minimum exposure to evidence-informed PTSD treatment recommendations. Mobile mental health apps, particularly those that are able to achieve such widespread reach, have unprecedented potential for improving quality of life and public health outcomes for those living with PTSD symptoms and other mental health conditions.

## Abbreviations

CBT: cognitive-behavior therapy
EULA: end user license agreement
PCL: PTSD check list
PTSD: posttraumatic stress disorder
SDK: software development kit
VA: Department of Veterans Affairs
